# Yield and nutrient augmentations in wheat: Application of plasma processed zinc

**DOI:** 10.1371/journal.pone.0343231

**Published:** 2026-02-25

**Authors:** Mamunur Rashid, M R Talukder, Monzur Hossain

**Affiliations:** 1 Plasma Engineering Lab, Department of Electrical and Electronic Engineering, University of Rajshahi, Rajshahi, Bangladesh; 2 Institute of Biological Sciences, University of Rajshahi, Rajshahi, Bangladesh; 3 Plant Breeding and Gene Engineering Lab, Department of Botany, University of Rajshahi, Rajshahi, Bangladesh; University of Technology and Applied Sciences - Salalah, OMAN

## Abstract

Billions of people worldwide suffer from hidden hunger caused by deficiencies in essential micronutrients, particularly zinc (Zn). Conventionally, Zn deficiency in humans has been addressed through dietary supplementation; however, agronomic biofortification offers a sustainable alternative. Plasma-assisted technology has recently emerged as a promising approach for enhancing Zn fortification in crops, although the underlying mechanisms governing Zn uptake and assimilation remain insufficiently understood. The present study investigates the mechanisms involved in plasma-mediated Zn fortification and its effects on plant physiology. Field experiments were conducted on wheat (*Triticum aestivum* L.) using plasma-processed ZnO, ZnSO₄, and their mixture (ZnO + ZnSO₄). A concentration of 200 ppm ZnO (ZnOTFS), ZnSO₄ (ZnSO₄TFS), and their mixture ((ZnO + ZnSO₄)TFS) was prepared in water and subsequently treated using plasma to generate plasma-processed water (PPW). The resulting solutions were applied as foliar sprays at an early growth stage of wheat plants. Growth parameters and carotenoid concentrations showed significant enhancement in plants receiving plasma-treated foliar applications compared to controls. Moreover, plasma-assisted foliar spraying reduced oxidative stress and significantly enhanced antioxidant enzyme activities, including catalase (CAT), ascorbate peroxidase (APX), and superoxide dismutase (SOD), in both leaves and roots. The concentrations of Zn and Fe in plant tissues increased by 50.35% and 28.81%, and by 94.30% and 62.34%, respectively, compared to the control and non-plasma-treated (ZnO + ZnSO₄) foliar spray treatments. In addition, grain quality and yield improved by 59%, 34%, and 26% relative to the control, PPW alone, and non-plasma-treated (ZnO + ZnSO₄) foliar spray treatments. These results demonstrate that plasma-assisted Zn fortification significantly enhances micronutrient accumulation, antioxidant defence, and yield attributes in wheat, highlighting plasma technology as a viable and sustainable strategy for combating micronutrient malnutrition.

## Introduction

Wheat (*Triticum aestivum* L.) is the widely edible crop after rice (*Oryza sativa* L.) and maize (*Zea mays* L.) in the Indian subcontinent [1,2]. Compared to other crops, it provides more calories, including proteins, to human beings [3] and animals. Different burning issues such as climate change, natural catastrophes and soil fertility have impacted on crop production chain [1]. Micronutrient fertilizers can address the deficiency of soil nutrients that contribute to increase crop productivity [4]. Proper supply of Zn in plants may increase the crop productivity as well as increase the accumulation of Zn in the edible parts of the plants. It is indispensable for numerous physiological functions in plants, such as the enhancement of enzymatic and metabolic activities, amino acid synthesis, and the production of chlorophylls. According to the study of Du et al. [5], ZnO can sometimes be more effective than ZnSO_4_ in increasing grain yield, biomass and Zn accumulation in several crops including wheat and rice. Further, ZnO and ZnSO_4_ are applied topically and absorbed by the leaves and translocated throughout the plants and thereby culminating in their presence inside the grains [6].

A diet lacking in specific micronutrients results in malnutrition, also referred to as hidden hunger, which has harmful but often undetectable health effects. Over 2 billion people worldwide suffer from micronutrient deficiencies [7]. This micronutrient deficiency is brought on by having a diet with insufficient nutrient content and food diversity. Nutrition-specific and nutrition-sensitive techniques can be used to address hidden hunger [8]. Dietary interventions, such as diet diversification, nutrient supplements including fortification, are part of the nutrition-specific or direct approach. Conversely, the biofortification strategy is part of the nutrient-sensitive approach. Increasing the concentration and bioavailability of nutrients in the edible portion of plants is known as biofortification [9]. Improving the concentration of micronutrients in staple food can be a long-term and economical way to improve human absorption of micronutrients [10,11]. The common food sources, particularly for underdeveloped nations, are cereal based crops [12,13]. Therefore, the supply of micronutrient-supplemented food grains can boost their intake of micronutrients. Staple foods including wheat, rice, and maize are fed on by a significant population worldwide, and hence biofortified Zn enriched food grains may greatly reduce the hidden hunger of the affected people.

The agricultural farm can take advantage of the new options offered by plasma technology to address the issues related to environmental sustainability, food security, and increased crop yields [14]. Atmospheric pressure cold (APC) plasma offers special advantages that can satisfy the specific agricultural farming needs. The potential applications of plasma processed water (PPW) in agriculture have been demonstrated by Rashid et al., [15]. When APC plasma discharge is exposed to water, reactive oxygen species (ROS) and reactive nitrogen species (RNS) are produced in water [16]. The primary concern of plasma agriculture is the enhancement of plant growth and yield. Seeds of different crops such as paddy [17], wheat [18], eggplant [19], and potatoes [20], were treated with APC plasma under different experimental conditions and consequently improved yields and nutritional quantities of crops. So far, no work has been reported about the effectiveness of plasma activated nutrient delivery in the field-level applications to the wheat plants and its particular impact on the bioavailability of nutrients to the edible portion. A few studies [21–23] demonstrated that the plasma application improved mineral uptake by the plants although the majority of these works are limited to lab-oriented. However, mechanisms concerning the fortification of nutrients in wheat by plasma technology under field conditions are mostly unknown. Since the present work is devoted to revealing the mechanisms of plasma assisted nutrient fortification to the wheat plants and the optimization of treatments under field conditions. In our earlier works [24–26], the use of APC plasma technology improved Zn fortification in edible crops like rice, eggplant and potatoes.

This field work was planned to fortify plasma processed Zn in the wheat grains. Plasma processed ZnO, ZnSO_4,_ and ZnO + ZnSO_4_ solutions (hereafter will be named as ZnOT, ZnSO_4_T, and (ZnO + ZnSO_4_)T) were applied to the wheat plants in the vegetative growth stage. Additionally, these investigations aid in increasing the bioavailability of Zn for plant uptake and thereby distributed it throughout the tissues and stored it in the edible portion of the plants. Further, the effects of the foliar spray of ZnOT (ZnOTFS), ZnSO_4_T (ZnSO_4_TFS), (ZnO + ZnSO_4_)T ((ZnO + ZnSO_4_)TFS) on the growth matrix, antioxidant activities, secondary metabolites, Zn concentration in the wheat grain, and the yield were examined.

## 2. Materials and methods

### 2.1 Plasma reactor

**Fig 1:**
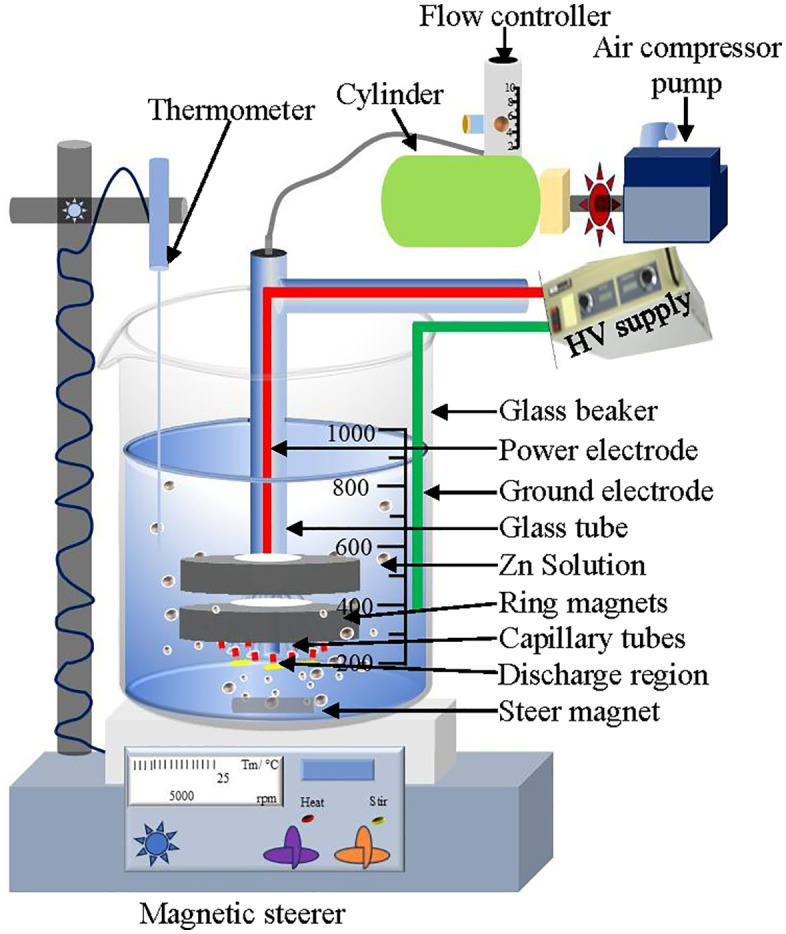
Schematic diagram of multi-capillary air bubble discharge plasma jet with circular magnet submerged in Zn solution.

A specially designed atmospheric pressure multi-capillary air bubble discharge cold plasma jet reactor, containing several jet tubes, was utilized for the preparation of PPW, ZnOT, ZnSO_4_T, and (ZnO + ZnSO_4_)T solutions to be foliar sprayed. Their concentrations were 200 ppm for ZnO and ZnSO_4_, while the concentrations of ZnO + ZnSO_4_ were (100 + 100) ppm. The optimum concentrations of ZnO and ZnSO_4_ applied in this investigation are considered to improve biofortification without causing toxicity [27]. In our earlier study, the reactor configuration was discussed [28], and the schematic diagram is presented in the Fig 1. Briefly, the reactor was made up of a 1.5 L borosilicate beaker, a high voltage power source (50 Hz, 10 kV, ~28W), two circular magnets, an air circulating chamber, and nine capillary tubes (length 15 mm, inside diameter 1 mm) for jet production. A plasma jet setup was immersed in ZnO, ZnSO_4_ and ZnO + ZnSO_4_ solutions, air was pushed through the array of jet tubes at a rate of 5 LPM. A ground wire was submerged in the solution and a power line was attached to the power electrodes of the capillary jets. Plasma jet was formed and the solutions were treated for 30 min. After 30 min treatment, the temperature of PPW remained at room temperature. The energy dissipated was $\text{2}.\text{7}\times {\text{1}\text{0}}^{\text{4}}$ J/L. The magnetic steerer was powered for spreading the plasma bubbles within the solution for a prolonged retention time to increase plasma bubble-water interaction. PPW was sprayed on the wheat plants within an hour after preparation.

### 2.2 Plasma species identification

The pH of the PPW, ZnO, ZnSO_4_ and ZnO + ZnSO_4_ solutions (with or without plasma treatment) was determined employing a pH meter following a 30 min treatment period. The optical method was utilized to measure the levels of ozone, hydrogen peroxide, nitrate and nitrite as discussed in our earlier study [29]. The densities of ${\text{H}}_{\text{2}}{\text{O}}_{\text{2}}$, ${\text{O}}_{\text{3}}$ ${\text{NO}}_{\text{2}}^{-}$ and ${\text{NO}}_{\text{3}}^{-}$ are listed in Table 1.

**Table 1:** pH, and the concentrations of $O_{3}$, $H_{2}O_{2}$, ${NO}_{2}^{-}$, and ${NO}_{3}^{-}$ in distilled water, PPW, plasma processed ZnO, ZnSO_4_ and their mixture prepared with magnetic field assisted atmospheric pressure multi-tube air bubble underwater discharge plasma jet. 1000 mL distilled water was treated at a time.

Condition	pH	$O_{3}{\;(mg.L}^{-1})$	$H_{2}O_{2}{\;(mg.L}^{-1})$	${NO}_{2}^{-}{\;(mg.L}^{-1})$	${NO}_{3}^{-}{\;(mg.L}^{-1})$
DI water	6.89	0	0	0	0
PPW	4.69	1.89	18.23	16.28	66.18
TZnO	4.35	2.10	20.28	18.32	69.15
TZnSO_4_	4.38	2.18	23.26	19.46	68.78
T (ZnO + ZnSO_4_)	4.23	2.27	24.35	24.39	71.25

### 2.3 Collection of seed

Wheat seed (*Triticum aestivum* L. BARI-32 GOM) was collected from the BWMRI (Bangladesh Wheat and Maize Research Institute, Rajshahi). Only healthy seeds were sown in the field after passing the visual screening process.

### 2.4 Field experiment

The field experiment was carried out in the agricultural research area of the Department of Electrical and Electronic Engineering, University of Rajshahi, Bangladesh. The land was prepared as per the requirements of the wheat field. Eight treatment conditions, including control, and foliar spray of PPW, ZnO, ZnSO_4_ and ZnO + ZnSO_4_ with and without plasma treatment, were assigned in subplots with three replications as part of the randomized complete block design trial. Considering that the number of unit plots was (7 × 3) +3 = 24, three control (untreated) plots were used to compare the results. The field size of each subplot was 10 m^2^. For sowing seeds, furrows were made properly using hand rakes. Seeds were sown continuously in line maintaining a depth of 2–3 cm from the soil surface. The line-to-line distance was kept at 20 cm. After sowing, the seeds were covered with soil. Irrigations were given at 20, 55 and 75 days after sowing (DAS) at the stage of root initiation, flowering and grain filling. Weeding was executed twice during the growing season: first at 20 DAS and second at 40 DAS. To maintain plant population density in each unit plot, the thinning process was done at 20 DAS. Foliar sprays of PPW and ZnOT, ZnSO_4_T and (ZnO + ZnSO_4_)T were carried out for four times (25, 35, 45 and 55 DAS) during their vegetative growth stage after 10-day interval.

### 2.5 Growth and yield study

Plant samples were randomly uprooted from the working area on different days after sowing (35, 70 and 105 DAS, respectively) to determine plant length (PL), stem diameter (SD), and weight of dried plants (DW) (at 70 DAS). PL and SD were measured using a scale, and the data were noted. An electronic balance was used to measure DW and the corresponding data were registered. Fresh leaves were assembled and ground with a mortar and pestle containing 90% methanol to estimate the concentrations of photosynthetic pigments such as chlorophyll a, chlorophyll b and carotene [30–32]. When the plant leaves and shoots became yellowish in color, then the crop was harvested separately from each plot. Panicle length, grain per panicle, 1000-grain weight and amount of grain per unit plot were measured and the corresponding data were recorded.

### 2.6 Antioxidant defense system

Randomly selected wheat plant samples were taken from the study field and washed with distilled water. 5000 µL of ${\text{KH}}_{\text{2}}{\text{PO}}_{\text{4}}$ solution (pH 7.0; 100 mM) was mixed with 100 mg of each tissue sample (leaves and roots) and ground using a mortar and pestle to make a homogenous mixture. The procedure of making the reaction mixture and the use of the uv-vis spectrometer to estimate the densities of CAT, APX, and SOD were provided in [33–35].

### 2.7 Determination of phenol

The method outlined in Ainsworth et al., [36] was used to measure the phenolic content in plant tissue. Randomly selected plant tissues (leaves and roots) were washed with distilled water and allowed to dry in the air. 0.20 g plant tissue were ground using 2 mL methanol (${\text{CH}}_{\text{3}}\text{OH}$) (5% v/v) using a mortar and pestle to make a homogenized mixture. After ten minutes at 12000 rpm centrifugation, the supernatant was placed into another microcentrifuge tube. The following chemicals: 50 µL plant extract, 250 µL of Folin-Ciocalteu (F-C) reagent, 0.50 mL deionized water, and 0.50 mL ${\text{NaCO}}_{\text{3}}$ (20% v/v) were added to prepare the reaction mixture. After combining chemicals gently, incubate the mixture for 30 minutes at room temperature. And thereafter, using the uv-vis spectrophotometer, the absorbance was measured at 765 nm and the phenol content was determined by comparing with the standard curve.

### 2.8 Determination of soluble sugar (SS) and protein (SP)

Using a spectrophotometer, concentrations of SS and SP in the plant tissue (leaves and roots) and in wheat grain were determined. Standard procedures, as described in [37], were followed in this study to estimate the SS and SP. In order to extract protein, plant tissues were pulverized by mixing the respective buffer solution (Methanol, 90%). Centrifuged the reaction mixture at 4000 rpm for 30 min and collected the supernatant into several microtubes. Optical densities were measured using a spectrophotometer, and hence estimated the protein concentrations in relation to the standard one. Plant samples were crushed using a mortar-pestle with 80% ethanol, and centrifuged at 10000 rpm for 10 min at an ambient for the estimation of SP concentration. The procedure, to determine SS concentration, for the preparation of the reaction mixture was followed as described in [38]. 0.10 mL of tissue supernatant mixed with 1000 µL of anthrone reagent and incubated at an ambient. The density of sugar in the extract was calculated in relation to the standard curves by measuring the absorbance at 620 nm.

### 2.9 Estimation of mineral

Wheat grains were cleaned, dehydrated at 70°C for 8 h, grounded into powder and then put into a crucible. The sample containing crucible was put in a muffle furnace, and the furnace temperature was raised steadily to 550°C for 6 h to make gray white ash. Adding 200 mg ash with 10 mL of HCl (0.05M) and centrifuge it at 6000 rpm for three min, then filtered the mixture. For measuring the absorbance, a flame atomic absorption spectrometer (AAS) was employed, and the corresponding mineral concentrations were obtained by calibrating with a standard reference.

### 2.10 Determination of proximate nutrients

Wheat grain powder was dried in a drying oven at 105°C until a consistent weight was reached in order to assess the moisture content [39]. The AOAC method was employed to measure ash content in triplicate of the samples. where the samples were burned in a muffle furnace at 600°C for eight hours [40]. The micro-kjeldahl method, as outlined by AOAC [41], was used to calculate the protein content. Firstly, nitrogen content was determined and the result was translated into percentage (%) of crude protein by multiplying by 6.25. Using the Soxhlet device, total lipid content of sample was ascertained [42]. By using Acid-Alkali Hydrolysis, which was followed by AOAC, crude fiber was identified [43]. The difference between the values of moisture, ash, protein, fat and crude fiber was used to calculate the amount of available carbohydrate [44]. By determining the amounts of protein, fat, and carbohydrates of the food item considered and the energy content of the sample was estimated by applying the following formula [45].


Energy=(Protein×4.1)+(Fat×9.3)+(Carbohydrate×4.1)

### 2.11 Statistical analysis

The experimental data obtained from the three replications were taken into account in the statistical analysis procedure. SPSS statistics 20 was used to analyze the experimental data. Duncan’s Multiple Range Test (DMRT) with a significance threshold of p < 0.05 was employed in the study along with a one-way ANOVA. The figures were prepared using Microcal Origin 21.

## 3. Results

### 3.1 Effect of plasma processed ZnO and ZnSO_4_ on growth characters

**Fig 2:**
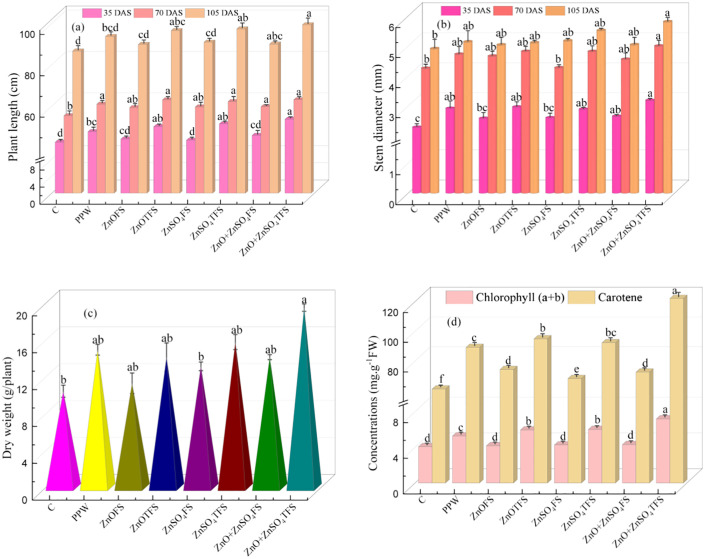
Effects of plasma processed ZnO and ZnSO_4_ foliar spray on (a) Plant length (cm), (b) Stem diameter (mm) (measured at 35, 70 & 105 DAS), (c) Dry weight (g/plant) and (d) Chlorophyll and carotene concentrations (measured at 75 DAS). Error bar indicates standard error of the replications and letters represent statistically significant differences at p ≤ 0.05. Meaning of the horizontal axis: C: control plants; PPW: Plasma Processed Water; ZnOFS: ZnO foliar spray without plasma treatment; ZnOTFS: ZnO foliar spray with plasma treatment; ZnSO_4_FS: ZnSO_4_ foliar spray without plasma treatment, ZnSO_4_TFS: ZnSO_4_ foliar spray with plasma treatment; (ZnO + ZnSO_4_)FS: (ZnO + ZnSO_4_) foliar spray without plasma treatment; (ZnO + ZnSO_4_)TFS: (ZnO + ZnSO_4_) foliar spray with plasma treatment.

Fig 2 shows the consequences on PL, SD, DW (at 75 DAS) and the densities of chlorophyll a, b and carotene (at 75 DAS) due to four-time applications of foliar spray of ZnO, ZnSO_4_, and ZnO + ZnSO_4_. The symbols used in horizontal axis indicate C: control, PPW: plasma processed water, ZnOFS: untreated ZnO foliar spray (FS), ZnOTFS: plasma treated ZnO foliar spray, ZnSO_4_FS: untreated ZnSO_4_ foliar spray, ZnSO_4_TFS: plasma treated ZnSO_4_ foliar spray, (ZnO + ZnSO_4_)FS: untreated (ZnO + ZnSO_4_) foliar spray, and (ZnO + ZnSO_4_)TFS: plasma treated (ZnO + ZnSO_4_) foliar spray. At 35, 70, and 105 DAS, the plants treated with (ZnO + ZnSO_4_)TFS exhibited maximum PL of 53.62, 63.05, and 99.33 cm, respectively. The improvements of PL in (ZnO + ZnSO_4_)TFS treated plants at 105 DAS were 14.61%, 6.05% and 10.37% respectively, in comparison to control, PPW and (ZnO + ZnSO_4_)FS treated plants, as shown in Fig 1(a).

According to Figs 2(b) and 2(c), the plants treated with (ZnO + ZnSO_4_)TFS exhibited the largest SD and DW, measuring 5.80 mm, and 19.49 g/plant, respectively. The SD and FW in (ZnO + ZnSO_4_)TFS were increased in 18.29% and 86.96%, 13.06% and 30.59%, and 15.19% and 37.99% respectively, in accordance with control, PPW and (ZnO + ZnSO_4_)FS treated plants. Plants treated with (ZnO + ZnSO_4_)TFS showed a maximum total chlorophyll concentration of 7.19 mg.g^-1^.FW and their enhancement were 76.95%, 36.39% and 67.01% respectively, shown in Figs 1(d), compared to control, PPW and (ZnO + ZnSO_4_)FS treated plants. Further, the highest carotene content was 122.11 mg.g^-1^.FW found in (ZnO + ZnSO_4_)TFS treated plants, which is 99.72%, 36.95% and 68.38% respectively, higher than that of control, PPW and (ZnO + ZnSO_4_)FS treated plants.

### 3.2 Effect of plasma processed ZnO and ZnSO_4_ on defense system

**Fig 3:**
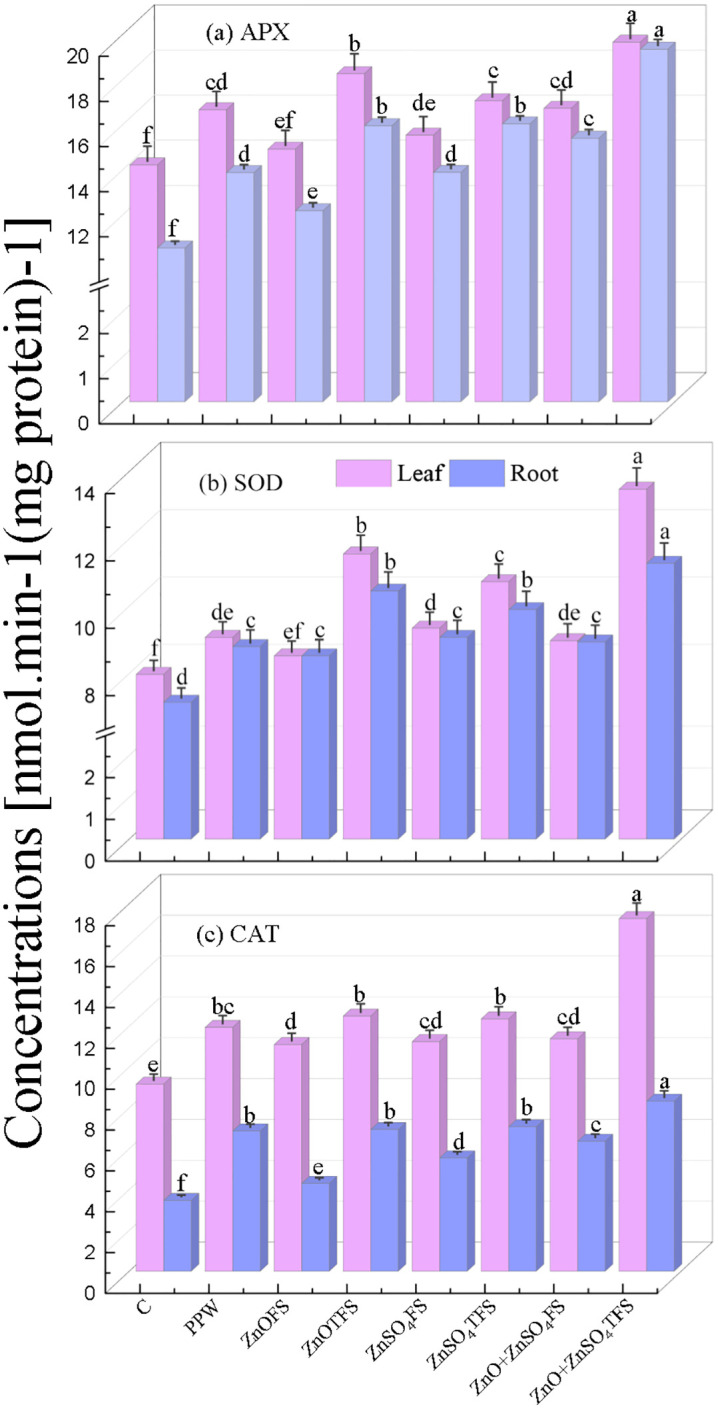
Effect of plasma processed ZnO and ZnSO_4_ foliar spray on (a) Ascorbate peroxidase (APX) (b) Superoxide dismutase (SOD), (c) Catalase (CAT) (measured at 75 DAS) in leaves and roots of wheat plants. Error bars indicate the standard error of the replications and letters represent statistically significant differences at p ≤ 0.05. X-axis symbols meanings are same as in Fig 2.

As illustrated in (Fig 3a-3c), the combined effects of plasma processed ZnO, ZnSO_4_ and (ZnO + ZnSO_4_) foliar application on concentrations of APX, SOD and CAT in leaves and roots are obtained at 75 DAS.

Fig 3(a) demonstrates that the maximum levels of APX concentrations in leaf and root, as measured from plants treated with (ZnO + ZnSO_4_)TFS, are 19.65 and 19.34 nmol.min^-1^.(mg protein)^-1^. Their increments are 38.80% and 83.71%, 17.99% and 39.45%, and 17.53% and 25.66% correspondingly, in terms of control, PPW and (ZnO + ZnSO_4_)FS treated plants. The highest SOD activity in the leaves and roots of plants treated with (ZnO + ZnSO_4_)TFS are 13.475 and 11.28 nmol.min^-1^.(mg protein)^-1^ as shown in Fig 3(b). The increase in SOD in leaf and root is 68.97% and 57.69%, 48.48% and 28.13%, and 50.14% and 26.12% respectively, higher than that of control, PPW and (ZnO + ZnSO_4_)FS treated plants. Further, as illustrated in Fig 3(c), the plants treated with (ZnO + ZnSO_4_)TFS exhibit the highest levels of CAT activity in their leaves and roots measuring 17.27 and 8.33 nmol.min^-1^.(mg protein)^-1^ respectively. CAT enhanced in leaf and root by 88.49% and 140.02%, 44.65% and 21.21%, and 51.72% and 30.99% respectively, in comparison to control, PPW and (ZnO + ZnSO_4_)FS treated plants. CAT activity is observed greater in leaf than in root.

### 3.3 Effect of plasma processed ZnO and ZnSO_4_ on phenol

**Fig 4:**
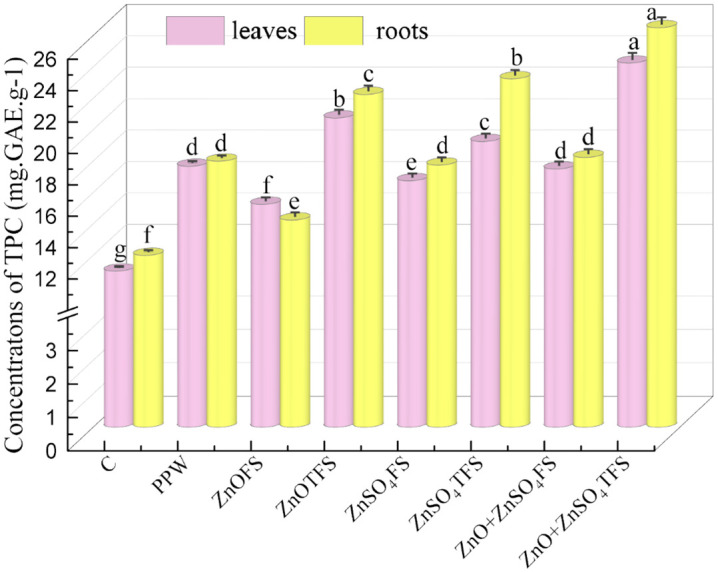
Effect of plasma processed ZnO and ZnSO_4_ foliar spray on total phenolic content (TPC) (measured at 75 DAS) in leaves and roots of wheat plants. Error bars indicate the standard error of the replications and letters represent statistically significant differences at p ≤ 0.05. X-axis symbols meanings are same as in Fig 2.

The collective effects of plasma processed ZnO, ZnSO_4_, and (ZnO + ZnSO_4_) foliar application on the amounts of TPC in the roots and leaves are presented in Fig 4. Leaf contains a maximum of 24.25 mg.GAE.g^-1^ of TPC when plants received (ZnO + ZnSO_4_)TFS treatment, and the enhanced concentration of TPC compared to control, PPW and (ZnO + ZnSO_4_)FS treated plants are 120.45%, 65.25% and 38.57% respectively. Furthermore, highest TPC in root is 26.50 mg.GAE.g^-1^ exhibit where (ZnO + ZnSO_4_)TFS treatment was provided. TPC in (ZnO + ZnSO_4_)TFS treated roots is increased by 140.91%, 49.93% and 51.48% respectively, in accordance with control, PPW and (ZnO + ZnSO_4_)FS treatments.

### 3.4 Effect of plasma processed ZnO and ZnSO_4_ on soluble sugar and protein content

**Fig 5:**
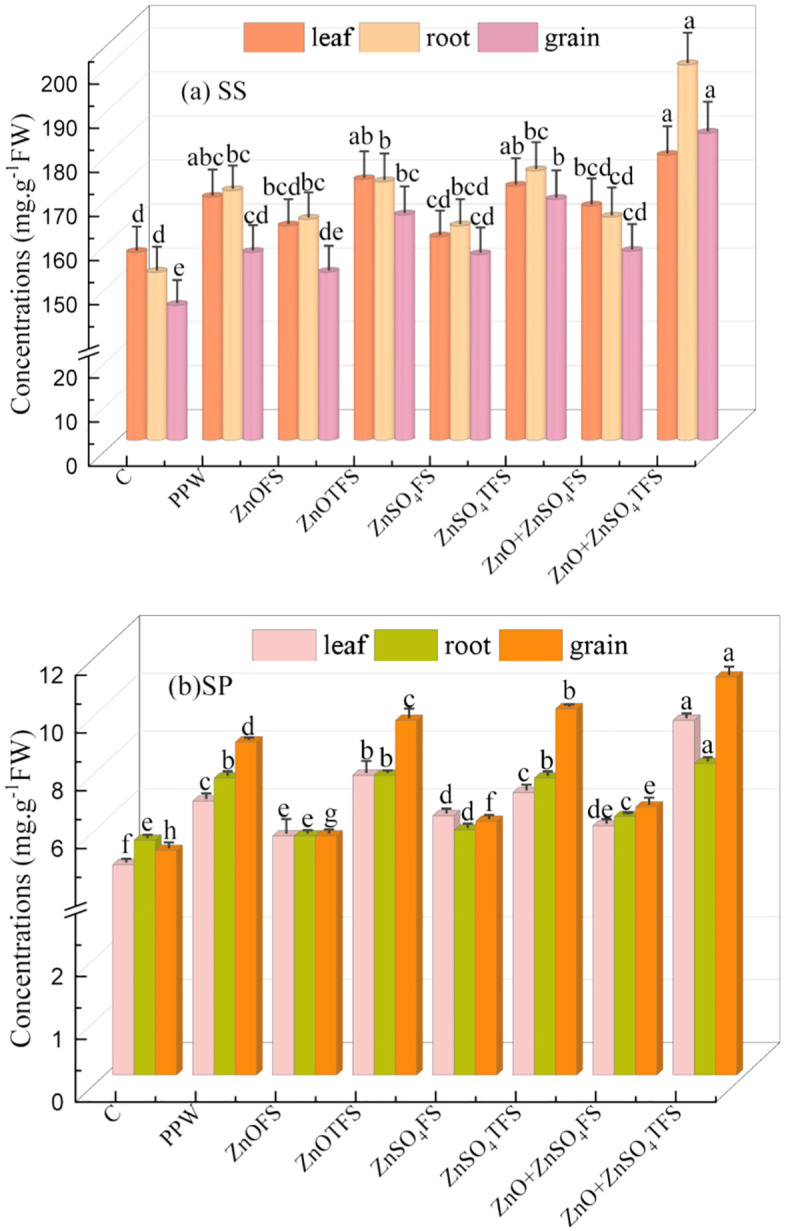
Effect of plasma processed ZnO and ZnSO_4_ foliar spray on (a) soluble sugar (SS) and (b) soluble protein (SP) (measured at 105 DAS) in leaves, roots and grain of wheat plants. Error bars indicate the standard error of the replications and letters represent statistically significant differences at p ≤ 0.05. X-axis symbols meanings are same as in Fig 2.

Fig 5(a) and 5(b) present the collective effects of plasma processed ZnO, ZnSO_4_, and (ZnO + ZnSO_4_) spray on the densities of SS and SP. SS densities in the leaves, roots and grains of the plants treated with (ZnO + ZnSO_4_)TFS were the highest, measuring 178.00, 198.50, and 183.00 mg.g^-1^ FW, respectively. Comparison shows that the SS content in (ZnO + ZnSO_4_)TFS plants increases by 27.08%, 17.31% and 17.12% respectively in wheat grains than control, PPW and (ZnO + ZnSO_4_)FS treated plants. Further, Maximum SP densities of 9.50, 8.00 and 11.00 mg. g^-1^ FW are found in the leaves, roots and produced grains in the plants treated with (ZnO + ZnSO_4_)TFS. Protein levels in wheat grains are elevated by 120%, 25.71% and 69.23% respectively, in comparison to control, PPW and (ZnO + ZnSO_4_)FS treated plants.

### 3.5 Effect of plasma processed ZnO and ZnSO_4_ on minerals

**Fig 6:**
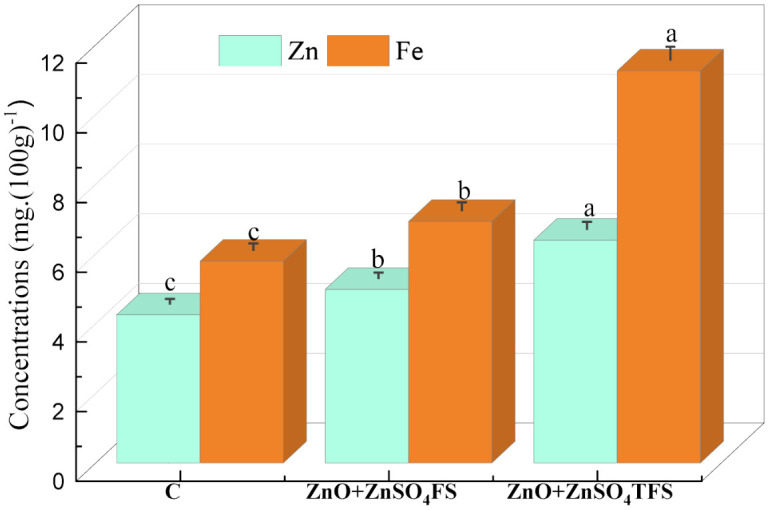
Effect of plasma processed ZnO and ZnSO_4_ spray on the concentration of Zn and Fe in wheat grains. Error bars indicate the standard error of the replications and letters represent statistically significant differences at p ≤ 0.05. X-axis symbols meaning same as Fig 2.

Fig 6 shows the combined consequences of plasma processed ZnO, ZnSO_4_ and ZnO + ZnSO_4_ spray on the mineral content especially Zn and Fe in wheat flour. Maximum Zn content was 6.39 mg. (100g)^-1^ found where (ZnO + ZnSO_4_) TFS was applied. Level of Zn was enhanced by 50.35% and 28.81% respectively, in accordance with control and (ZnO + ZnSO_4_)FS treatments. Further the highest Fe content was 11.25 mg. (100g)^-1^ found in the (ZnO + ZnSO_4_)TFS treated plants with the increments of 94.30% and 62.34% respectively, in accordance with control and (ZnO + ZnSO_4_)FS treated plants.

### 3.6 Effect of ZnO and ZnSO_4_ on the proximate nutrients

**Fig 7:**
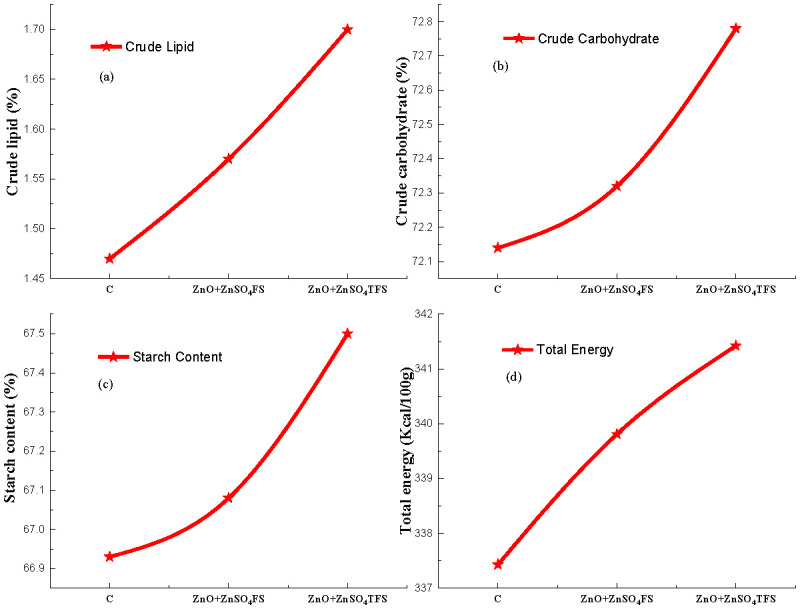
Effect of plasma processed ZnO and ZnSO_4_ foliar spray on (a) crude lipid (%), (b) crude carbohydrate (%), (c) starch content (%) and (d) total energy (kcal/100g) in wheat grains. Error bar indicates standard error of the replicates and letter represent statistically significant differences at p ≤ 0.05. X-axis symbols meanings are same as in Fig 2.

Effects of plasma processed ZnO, ZnSO_4_ and (ZnO + ZnSO_4_) foliar sprays on the crude lipid (%), crude carbohydrate (%), starch content (%), and total energy (Kcal/100g) of wheat flour are depicted in (Fig 7a-7d). Maximum crude lipid, shown in Fig 7(a), is 1.70% found in the flour, produced from the (ZnO + ZnSO_4_)TFS treated plants. In comparison to control and (ZnO + ZnSO_4_)FS, the increments in lipid are 15.65% and 8.28% respectively. Fig 7(b) and 7(c) show the highest crude carbohydrate and starch content 72.78% and 67.50% in the grains where (ZnO + ZnSO_4_)TFS treatment was provided and their enhancements are 0.89% and 0.85%, and 0.64% and 0.63%, respectively, with respect to control and (ZnO + ZnSO_4_)FS. Further, the maximum total energy content in grains is obtained by 341.42(kcal/100g), in the flour produced from the (ZnO + ZnSO_4_)TFS treatment. Total energy contents in grains are elevated by 1.18% and 0.47%, respectively, compared to control and (ZnO + ZnSO_4_)FS treatments.

**Fig 8:**
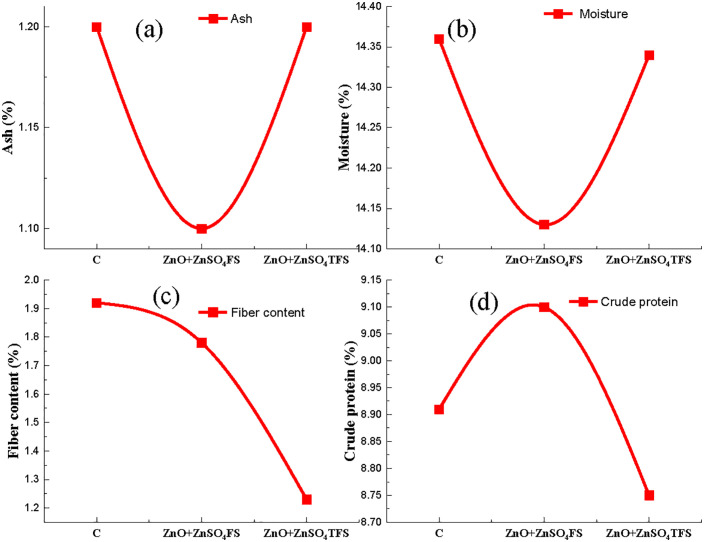
Effect of plasma processed ZnO and ZnSO_4_ foliar spray on (a) Ash (%), (b) Moisture (%), (c) Fiber content (%) and (d) Crude protein (%) in wheat grains. Error bar indicates standard error of the replications and letters represent statistically significant differences at p ≤ 0.05. X-axis symbols meaning same as Fig 2.

On the other hand, (Fig 8a-8d) displays the ash (%), moisture (%), fiber content (%) and crude protein (%) under the consequences of plasma processed ZnO, ZnSO_4_ and (ZnO + ZnSO_4_) spray to the wheat plants. Ash content in wheat flour, displayed in Fig 8(a), is equal to (ZnO + ZnSO_4_)TFS treatment and control whereas slightly lower in (ZnO + ZnSO_4_)FS. Maximum moisture content in flour is 14.36% found in the control, shown in Fig 8(b). It is decreased in (ZnO + ZnSO_4_)FS but increased in the (ZnO + ZnSO_4_)TFS treatment. Further, the maximum fiber content is 1.78% observed in the control while it is gradually decreased in the Zn treated grains, depicted in Fig 8(c). Furthermore, maximum crude protein is 9.1% obtained from the (ZnO + ZnSO_4_)FS treated grains, presented in Fig 8(d), where it is increased by 2.13% compared to control. But in the (ZnO + ZnSO_4_)TFS treated grains, it is slightly reduced by 3.85% with respect to (ZnO + ZnSO_4_)FS treatment.

### 3.7 Effect of ZnO and ZnSO_4_ on yield and yield related characters

**Fig 9:**
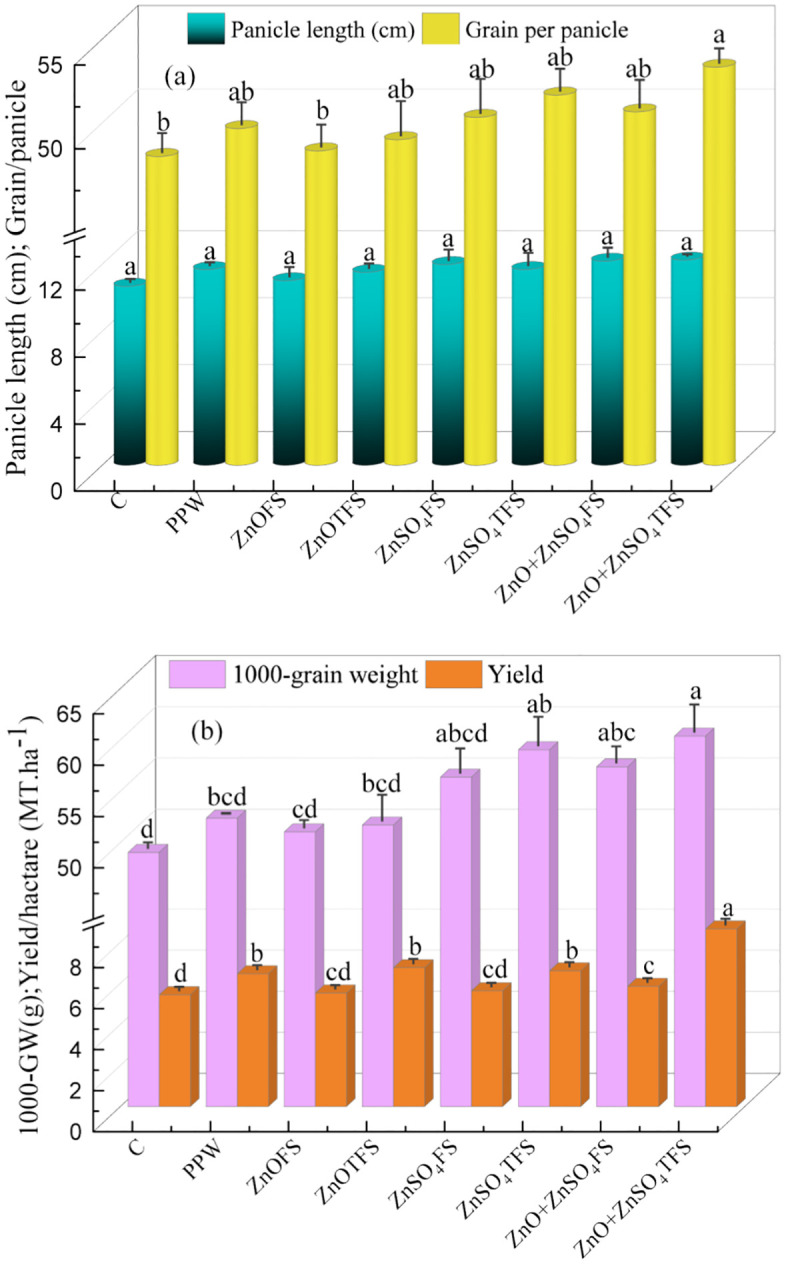
Effect of plasma processed ZnO and ZnSO_4_ foliar spray on (a) panicle length (cm) & grain per panicle and (b) 1000-grain weight (g) & yield (MT.ha^-1^) of wheat. Error bar indicates standard error of the replications and letter represent statistically significant differences at p ≤ 0.05. X-axis symbols meanings are same as in Fig 2.

The combined consequences of plasma processed ZnO, ZnSO_4_ and (ZnO + ZnSO_4_) sprays on the wheat yield and yield characters are shown in (Fig 9a-9b). The results reveal that the plants produced the longest panicle and highest number of wheat grains with a maximum of 12.26 cm and ~54 where (ZnO + ZnSO_4_)TFS treatment was provided. Compared to control, PPW and (ZnO + ZnSO_4_)FS with respect to (ZnO + ZnSO_4_)TFS treated plants, the improvements in panicle length and grains per panicle are 14.66% and 11.10%, 4.82% and 7.38%, and 0.55% and 5.26%, respectively. Besides, in comparison to control, PPW and (ZnO + ZnSO_4_)FS treated plants, the maximum 1000-grain weight of 60.39g was yielded in (ZnO + ZnSO_4_)TFS treated plants, with increments of 23.10%, 15.27% and 5.23% respectively. Alternatively, the plants treated with (ZnO + ZnSO_4_)TFS were provided a maximum of 8.66 MT.ha^-1^ and the yields were enhanced by ~59%, ~34% and ~26% respectively, compared to control, PPW and (ZnO + ZnSO_4_)FS treated plants.

## 4. Discussion

APC plasma usually produces RNS and ROS, with atomic oxygen anions (O^-^), superoxide anions (O_2_^-^), and hydroxyl radicals (OH^*^) in air discharge. When RONS species are immersed in water, the species interact with water molecules at the interface as well as in the bulk and produce species such as OH^*^, H_2_O_2_, O_3_, ${\text{NO}}_{\text{2}}^{-}$, ${\text{NO}}_{\text{3}}^{-}$, $\text{HN}{\text{O}}_{\text{2}}$ and NOOH.

The combined consequences of plasma processed ZnO, ZnSO_4_ and (ZnO + ZnSO_4_) sprays on plant growth parameters such as PL, SD, DW and the concentrations of total chlorophyll and carotene are depicted in Figs 3(a) to 3(d). The growth parameters improved when plasma processed (ZnO + ZnSO_4_) was applied at a concentration of 200 ppm for four times. Through foliar spray, the Zn from ZnO or ZnSO_4_ can accumulate in the leaves, making them a potentially effective source of Zn for plant metabolism [46,47]. According to a recent study by Read et al., [48], ZnO absorption by wheat and sunflower (*Helianthus annuus* L.) was primarily guided by the leaf cuticle, whereas particle coating did not affect Zn absorption and might even improve Zn resupply because of ZnO or ZnSO_4_ adherence to leaves. In addition, ZnO is also used as nano-fertilizer, which may be a more effective and gradual release of Zn than conventional fertilizers, which may also be a slower release of Zn than other sources [46,49]. Further, (ZnO + ZnSO_4_) foliar spray treated with plasma can improve Zn uptake and translocation, which increases photosynthetic efficiency and the generation of essential enzymes, hence promoting wheat development. Increased concentrations of enzymes such as ATPase and Rubisco are necessary for photosynthesis, and higher chlorophyll content as well as increased Zn availability [50]. ZnO is not soluble while ZnSO_4_ is more soluble and bioavailable. Plasma treatment may improve their ability to pass through the plant cuticle and supply Zn to vital cellular functions, which would ultimately raise plant height, stem diameter and biomass [51].

The synergetic impact of (ZnO + ZnSO_4_)TFS increased the activity of the antioxidant enzymes such as SOD, CAT and APX in wheat plants. Plant health and stress tolerance are enhanced by this coordinated enzymatic action, which efficiently scavenges ROS and lowers oxidative stress [51]. In the chloroplast, cytosol and mitochondria, SOD catalyzes the conversion of superoxide free radicals into H_2_O_2_ and O_2_, which are known as early defense against oxygen free radicals [52]. It is the primary antioxidant enzyme that protects plants against oxidative stress brought on by ROS, which is a significant scavenger of O_2_^*^ free radicals that are transformed into H_2_O_2_ and O_2_ by catalase and many peroxidases [53]. CAT can effectively degrade high H_2_O_2_ concentrations and lessen the harm caused by OH radicals produced from H_2_O_2_. Thus, in plant cells, CAT regulates the amount of hydrogen peroxide. Another enzyme involved in H_2_O_2_ elimination is APX, and this enzyme helps regulate minute variations in H_2_O_2_ concentrations [54].

(ZnO + ZnSO_4_)TFS enhances wheat phenol content by making the Zn more available, promoting the activation of antioxidant defense enzymes such as phenylalanine ammonia-lyase, and providing direct antioxidants effects, acting as non-enzymatic antioxidant themselves [55]. The combination of plasma-generated species and (ZnO + ZnSO_4_) induces stress in the plant, which in turn triggers a stronger antioxidant response [56]. This includes the increased synthesis of total phenols and flavonoids, which act as a potential non-enzymatic antioxidant, scavenging harmful free radicals [57].

(ZnO + ZnSO_4_)FS raises wheat’s levels of soluble sugar and protein because of plasma treatment, as it increases their bioavailability. This is explained by the fact that plasma discharge electrostatically charges Zn particles, preventing aggregation and growth. Besides, the plasma produced reactive species (electrons and radicals) may further passivate the surfaces and stop aggregation [58]. Smaller particle sizes are thus formed as a result of these flaws and modifications [59,60] and thereby increasing their accessibility for plant uptake. Further, when (ZnO + ZnSO_4_)FS were applied topically, nitrogen assimilating enzymes including glutamine synthetase and nitrate reductase, became more active, which boosted nitrogen synthesis, transport and the quantity of precursor amino acid [61]. This, in turn, increases photosynthetic activity, enzymatic performance which ultimately raises the amount of soluble sugar and protein in the wheat leaves, roots and grain.

The findings from the atomic absorption spectroscopic analysis point to the fact that the Zn in wheat grain was no longer present as ZnO, demonstrating that ZnO did not develop particular toxicity. In this study, it was found that the highest Zn and Fe concentrations in the plants where (ZnO + ZnSO_4_)TFS treatment was provided compared to (ZnO + ZnSO_4_)FS treated plants. Foliar application of Zn triggers the antioxidant activity, which in turn enables higher Zn accumulation in the plants and thereby translocation to the wheat grains [62]. Zn mostly coexists with phosphate and sulfur [63]. The primary form of Zn in wheat is Zn phosphate, which poses no risk, based on current experimental findings [6]. Dimkpa et al., [64] investigated the accumulation of Cu and Zn in wheat seedlings treated with CuO and ZnO nanoparticles (NPs) and they found that ZnO NPs were absent from the shoots, whereas CuO NPs were present. Further, Du et al., [65] examined the impacts of ZnO and TiO_2_ NPs on wheat, and found that ZnO NPs were absent in the shoots but TiO_2_ NPs were present. According to their findings, ZnO NPs may be more soluble than CuO and TiO_2_ NPs, which could explain this occurrence. Besides, the level of Fe in wheat grains is increased synergistically due to the enhancement of Zn. Zn stimulates the physiological mechanisms of the plants that increase other nutrient uptake and translocation to the grain. Further, Zn increases the translocation of Fe by facilitating the transfer of nutrients stored in the vegetative portions during the grain filling stage [66].

Endogenous lipids, found naturally in wheat flour, are essential for dough development. Polar lipids, like lyso-phosphatidyl-ethanolamine (LPE) and phosphatidic acid (PA) as well as the free fatty acid (FFAs) produced when enzymes like lipases hydrolyze other lipids, are the main cause of degradation of the crude lipid of wheat flour [67]. According to Chen et al., [68], decreased lipase activity may promote lipid buildup, exacerbate oxidative stress and perhaps lead to lipid peroxidation. In this study, (ZnO + ZnSO_4_)TFS enhanced Zn uptake by wheat plants. Although Zn is a necessary cofactor for several enzymes but wheat lipase is inhibited by it. According to research [69], Zn can effectively suppress wheat lipase rather than activate it due to the fact that they bind to the enzyme and change its structure and activity. Foliar sprays, especially those that contain nitrogen and other micronutrients, enhance the effectiveness of photosynthetic processes and the transfer of nutrients to the grain where they are subsequently stored as carbohydrates [70]. Further, Zn plays a crucial role in several enzymes involved in carbohydrate [71] and sugar metabolism. Both of them are necessary for transforming sugar into starch and are decreased due to a shortage of Zn [72]. Starch makes up a significant portion of wheat and it is widely used in food and non-food industries. Granular form of amylose and amylopectin molecules comprises starch, which is found in nature in a range of 1–100 µm [73]. According to Chaiwat et al., [74], plasma treatment in one example of the green technology trend that has been used to alter starches without producing any trash. It can be seen in (Fig 8a-8d) that ash and moisture content are equal or near about the control and plasma Zn fortified plants, while decreasing in the plants where (ZnO + ZnSO_4_) FS was provided. But in fiber and crude protein contents, they gradually decrease because of Zn fortification with or without plasma treatment compared to the control. In particular, plasma can depolymerize fiber chains which increases the synthesis of soluble oligosaccharides and decreases the yield of fiber [75].

(ZnO + ZnSO_4_)TFS improved the physiological processes of the plant, including photosynthesis, antioxidant activity, water use efficiency and increased wheat production by increasing the Zn availability for plant uptake. Wheat yield can be increased when there is a positive association of Zn and nitrogen. Additionally, numerous enzymes involved in the synthesis of chlorophyll rely on nitrogen as a structural element, which controls plant growth, development and yield [76].

## Conclusion

Malnutrition, a barrier in reaching Sustainable Development Goal (SDG) including it’s all manifestations. It carries an enormous negative impact on both human well-being and nation’s economy. In order to achieve both food security and SDGs, a numerous research works are being done for fortifications of grain crops with micronutrients to maximize plants and better yields. The present study was designed to spray plasma processed ZnO + ZnSO_4_ to the wheat plants during their vegetative growth stage to optimize its effects on the plant growth matrix, physiological process, antioxidant activities, nutritional enrichment and yield. Plasma has the ability to increase the bioavailability of Zn for plant uptake, as well as influence the leaf cuticle for nutrient uptake. The findings show that Zn fortification applying plasma technology enhanced the plant defense system, nutrient content, Zn deposition in wheat grain and yield. Future large-scale application of plasma technology could transform crop fortification techniques by coordinating nutrient enrichment with agricultural efficiency. However, further investigation will be needed to understand the molecular characterization of Zn enrichment by applying plasma technology to the grain crops.

## Supporting information

S1 Table Basic data of Fig 2. (a) and Fig 2. (b). (JPG)

S2 Table Basic data of Fig 2. (c) and Fig 2. (d). (JPG)

S3 Table Basic data of Figs 3. (a), (b) and (c). (JPG)

S4 Table Basic data of Fig 4. (JPG)

S5 Table Basic data of Fig 5. (a) and Fig 5. (b). (JPG)

S6 Table Basic data of Fig 6. (JPG)

S7 Table Basic data of Figs 7. (a-d). (JPG)

S8 Table Basic data of Figs 8. (a-d). (JPG)

S9 Table Basic data of Figs 9. (a-b). (JPG)

S10 File Workflow. (TIF)
