# Supplementary figures and images for: Yield and nutrient augmentations in wheat: Application of plasma processed zinc
Source: PLoS One. 2026 Feb 25;21(2):e0343231. doi: 10.1371/journal.pone.0343231 (PMC12935264; doi:10.1371/journal.pone.0343231)

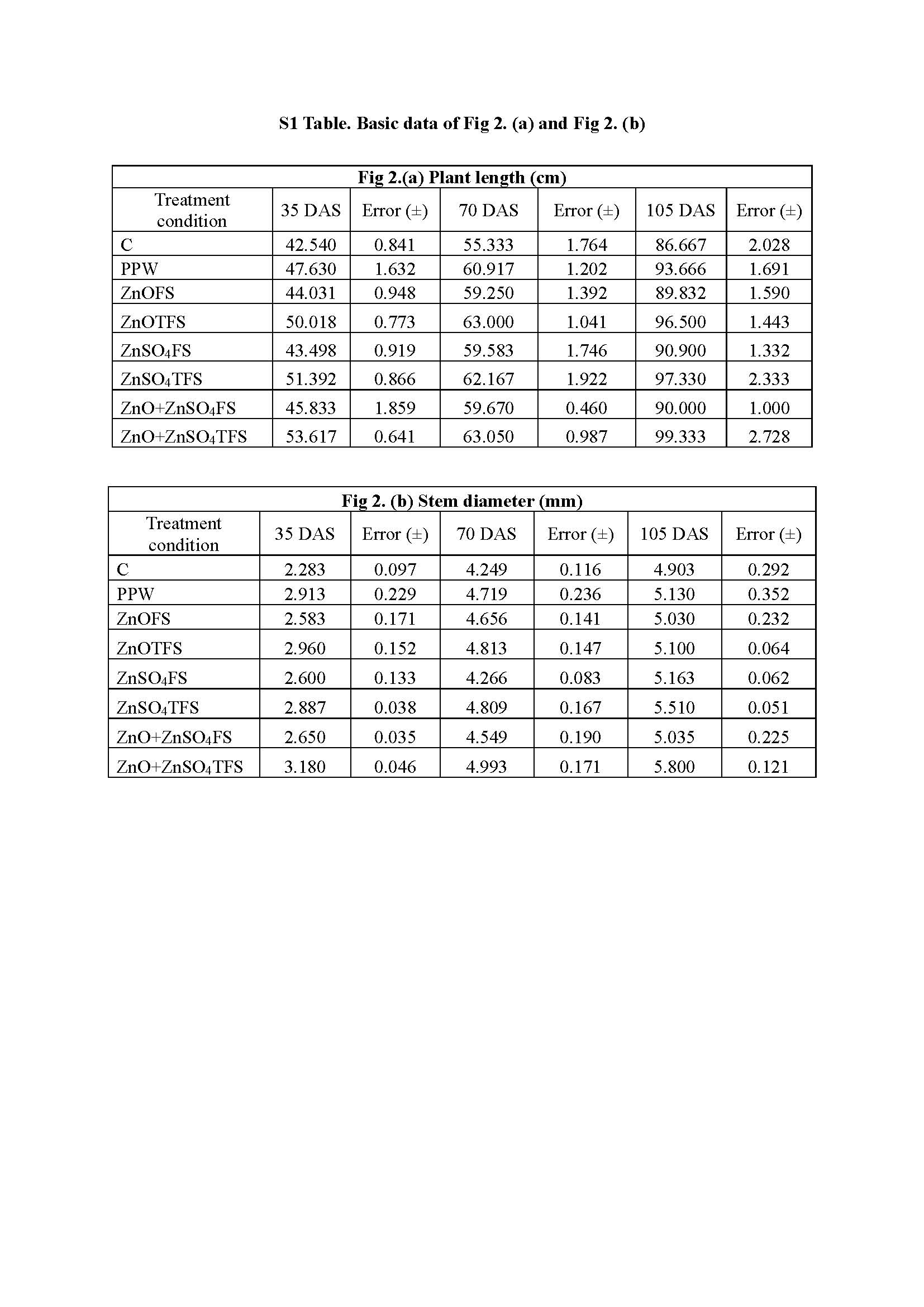

Supplement: S1 Table — (JPG) [file pone.0343231.s001.jpg]

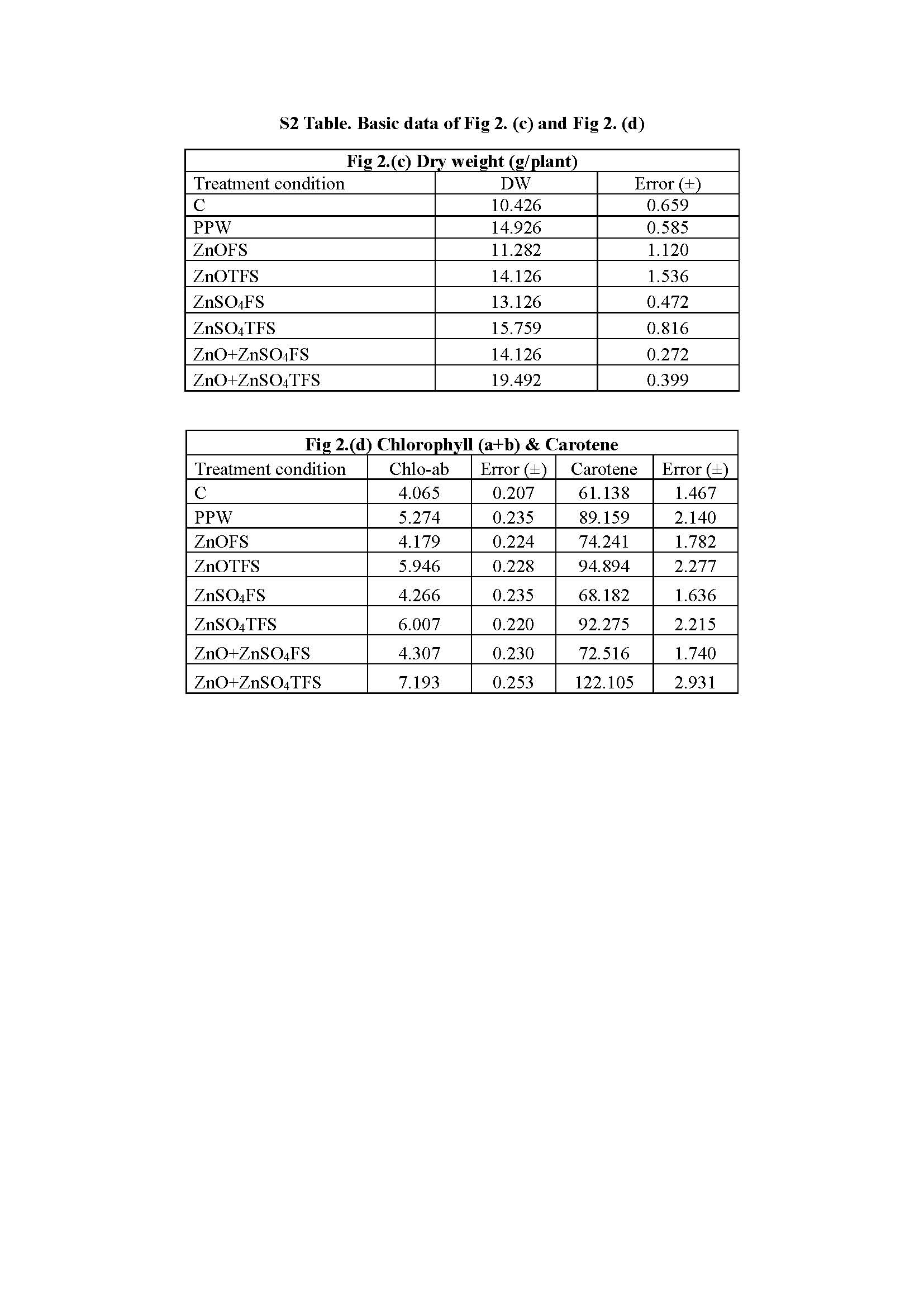

Supplement: S2 Table — (JPG) [file pone.0343231.s002.jpg]

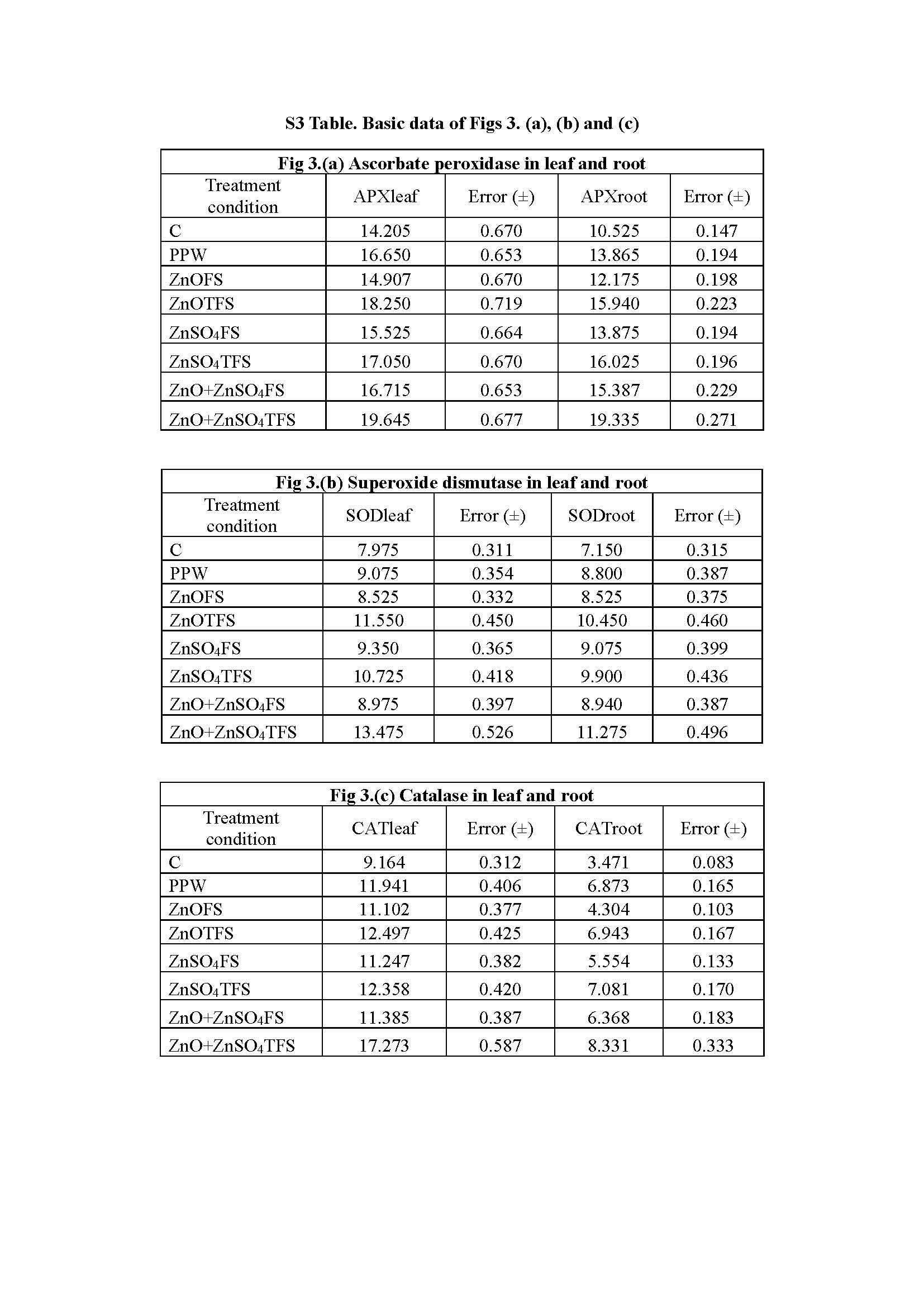

Supplement: S3 Table — (JPG) [file pone.0343231.s003.jpg]

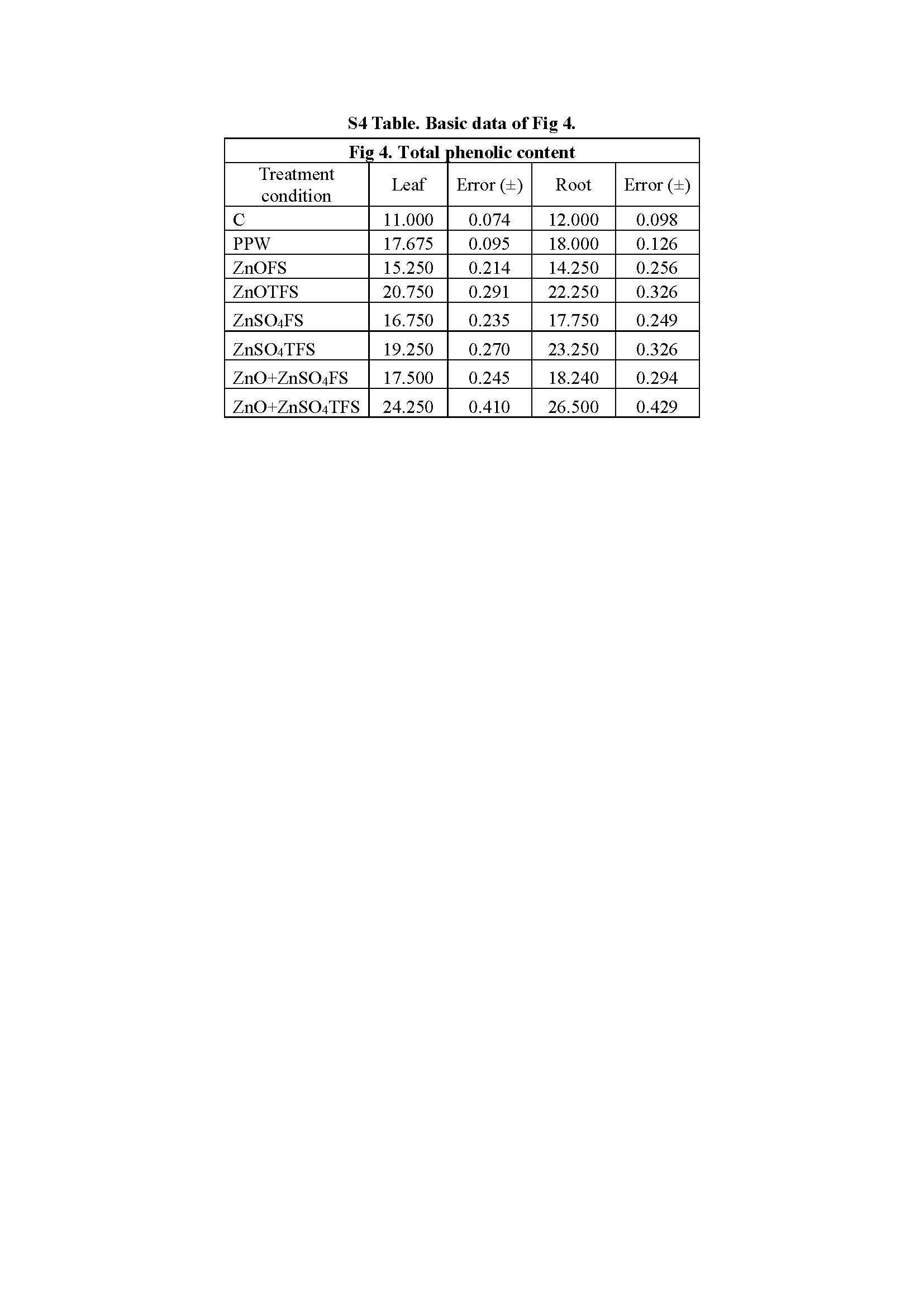

Supplement: S4 Table — (JPG) [file pone.0343231.s004.jpg]

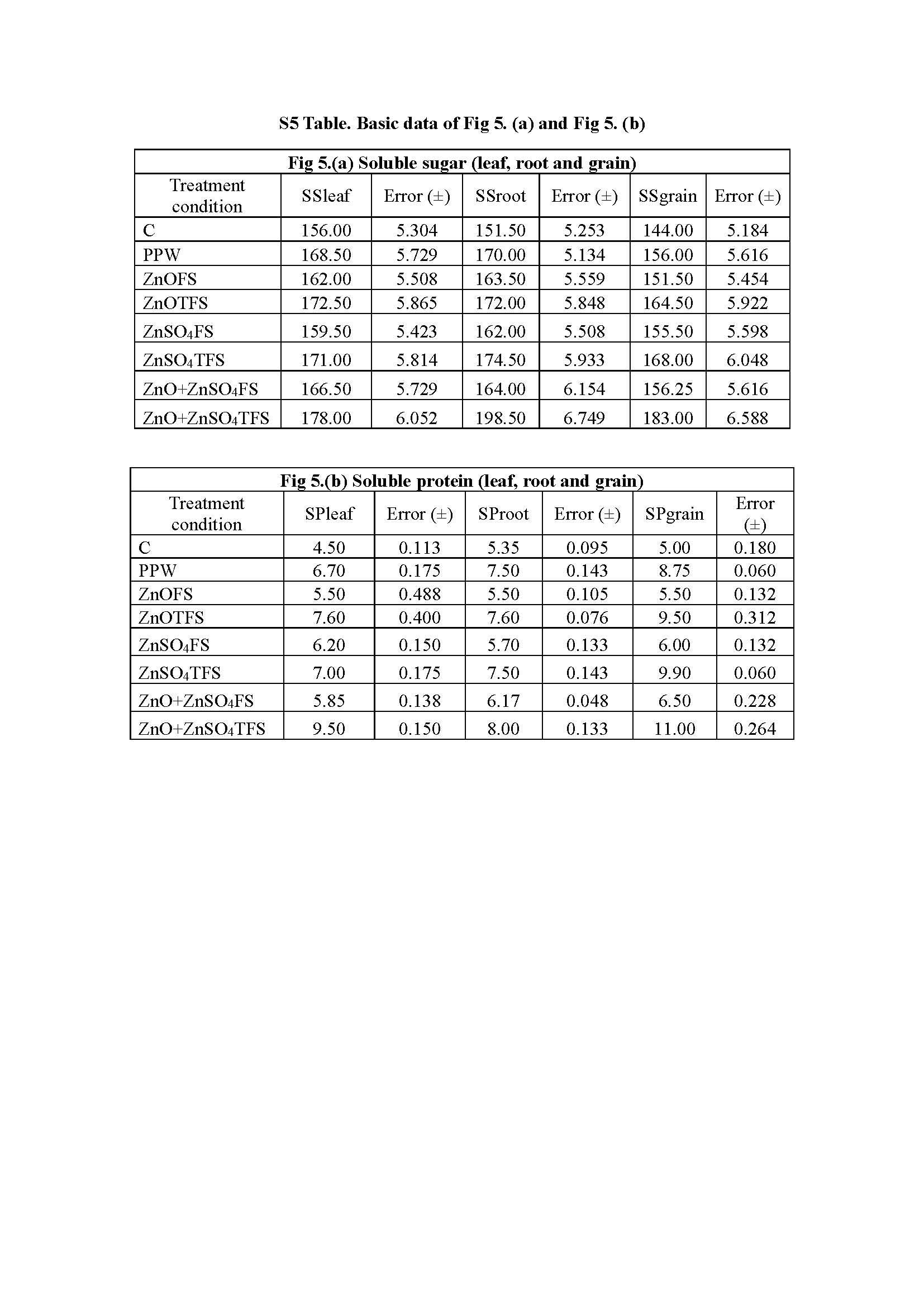

Supplement: S5 Table — (JPG) [file pone.0343231.s005.jpg]

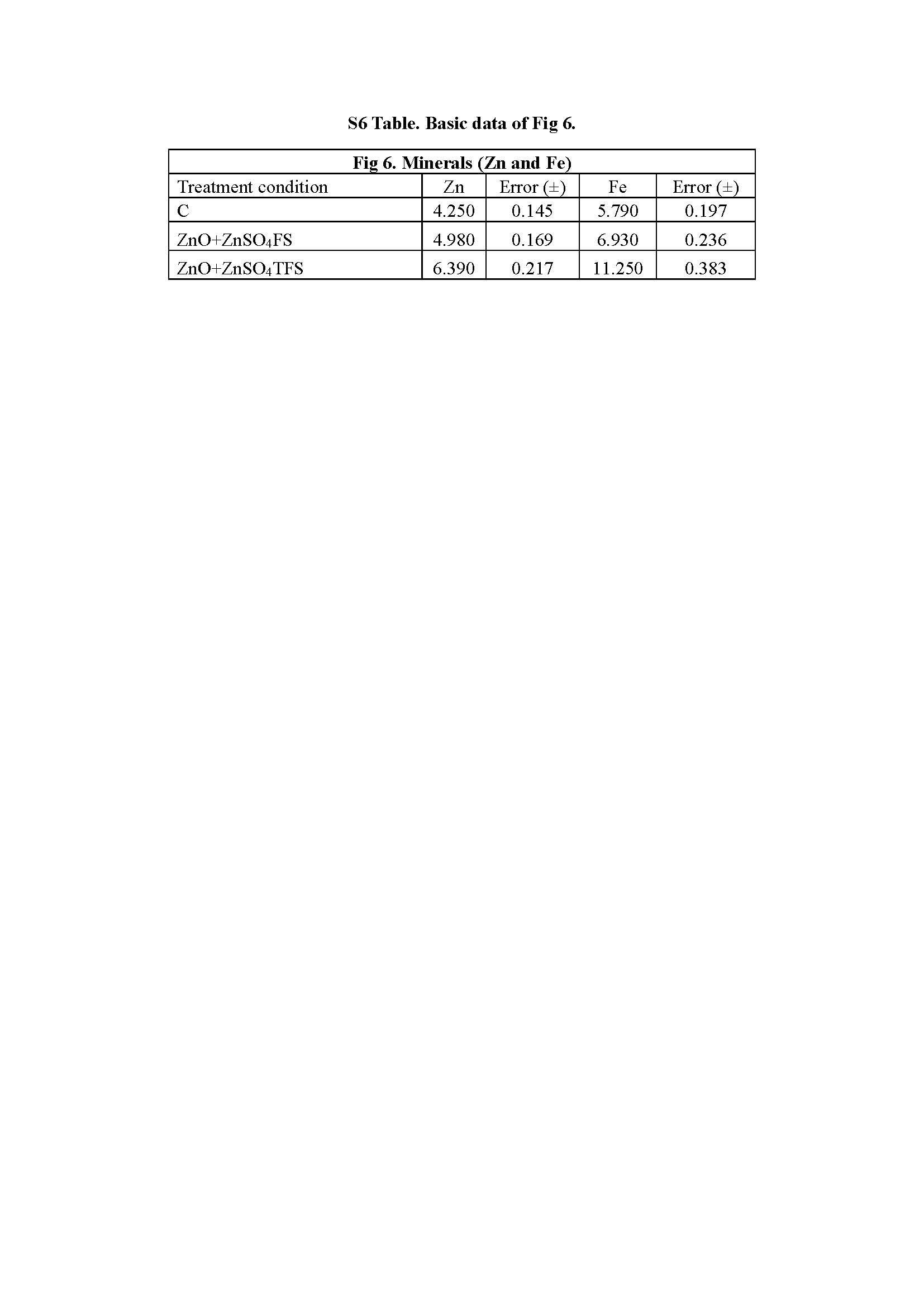

Supplement: S6 Table — (JPG) [file pone.0343231.s006.jpg]

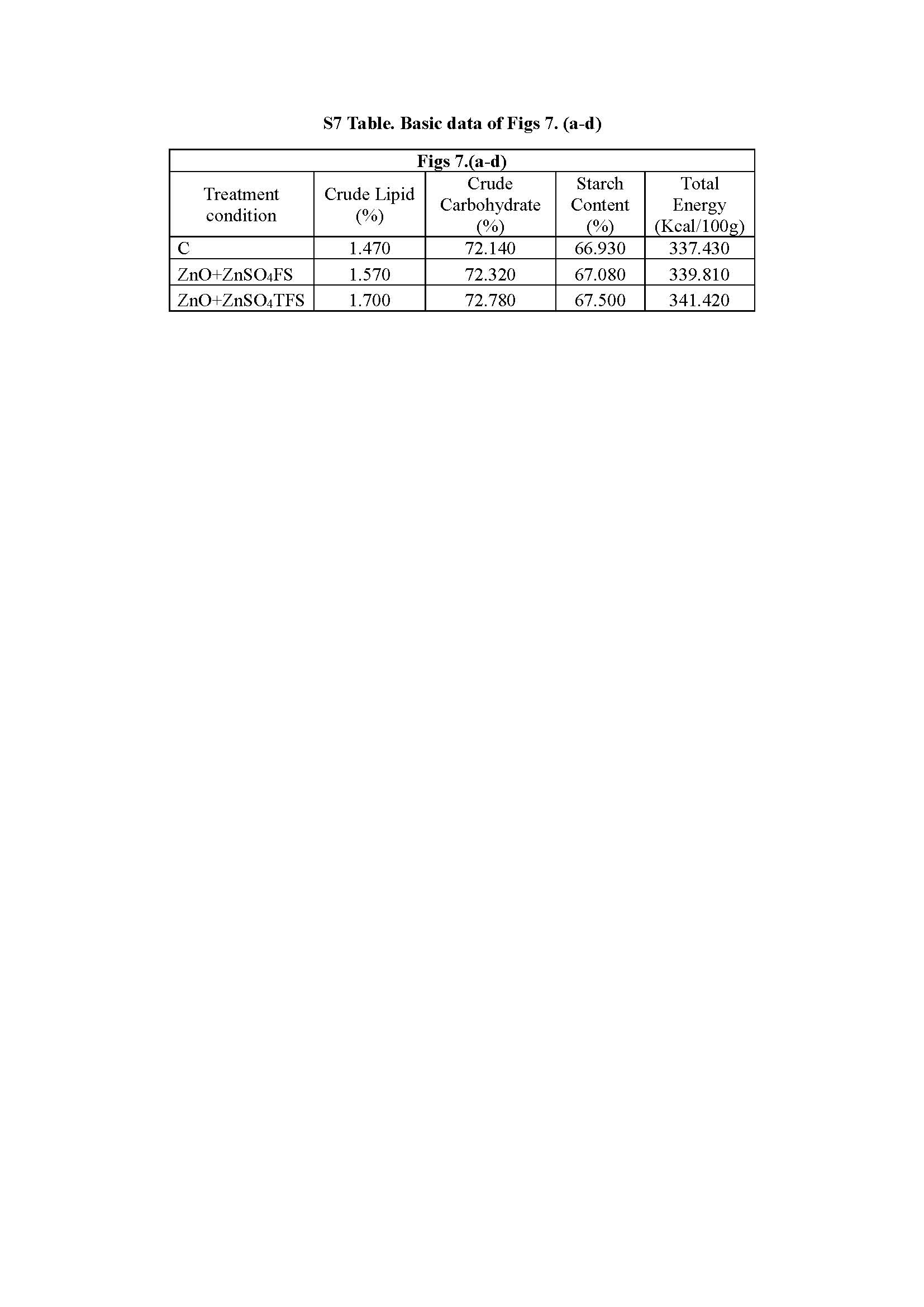

Supplement: S7 Table — (JPG) [file pone.0343231.s007.jpg]

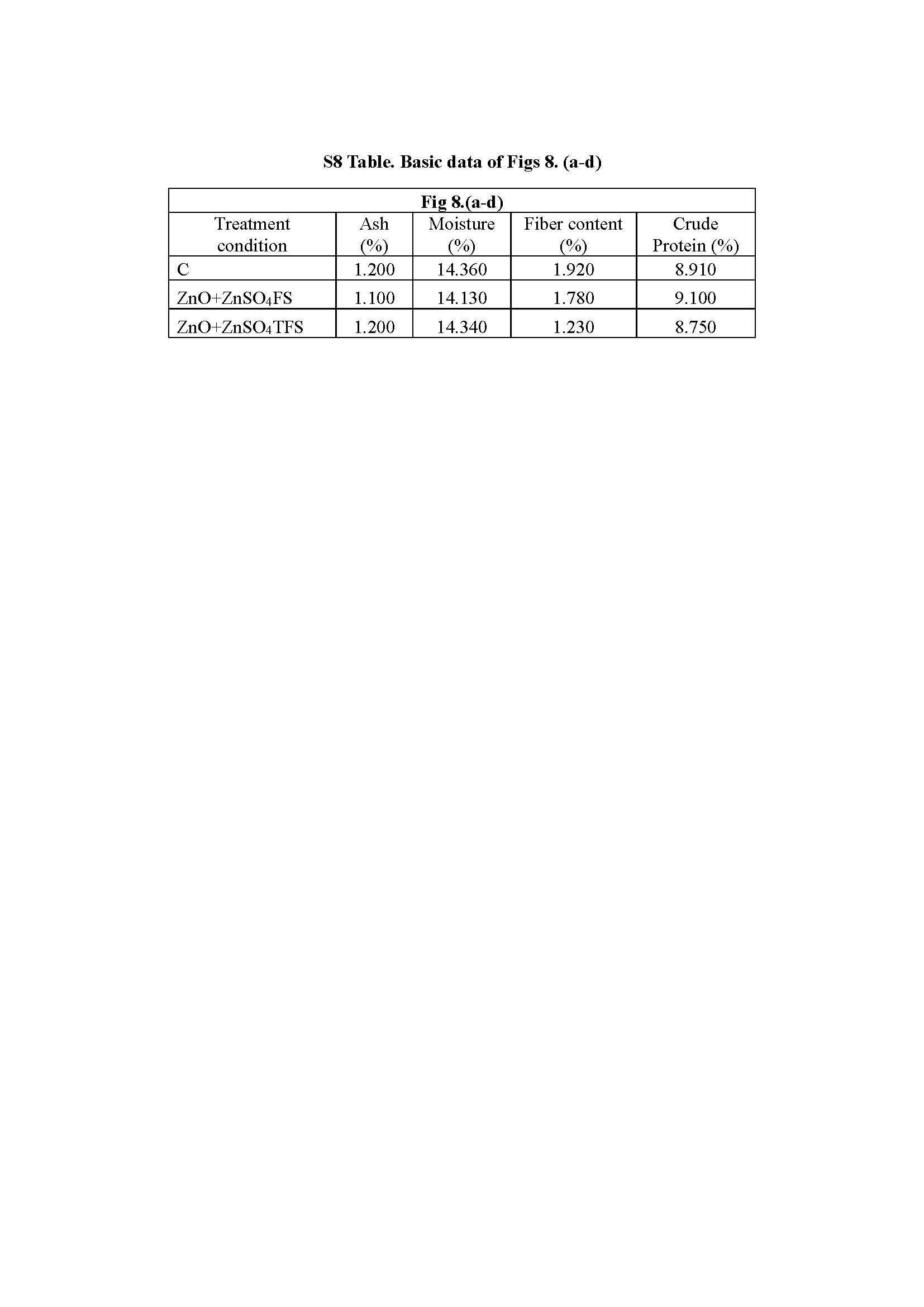

Supplement: S8 Table — (JPG) [file pone.0343231.s008.jpg]

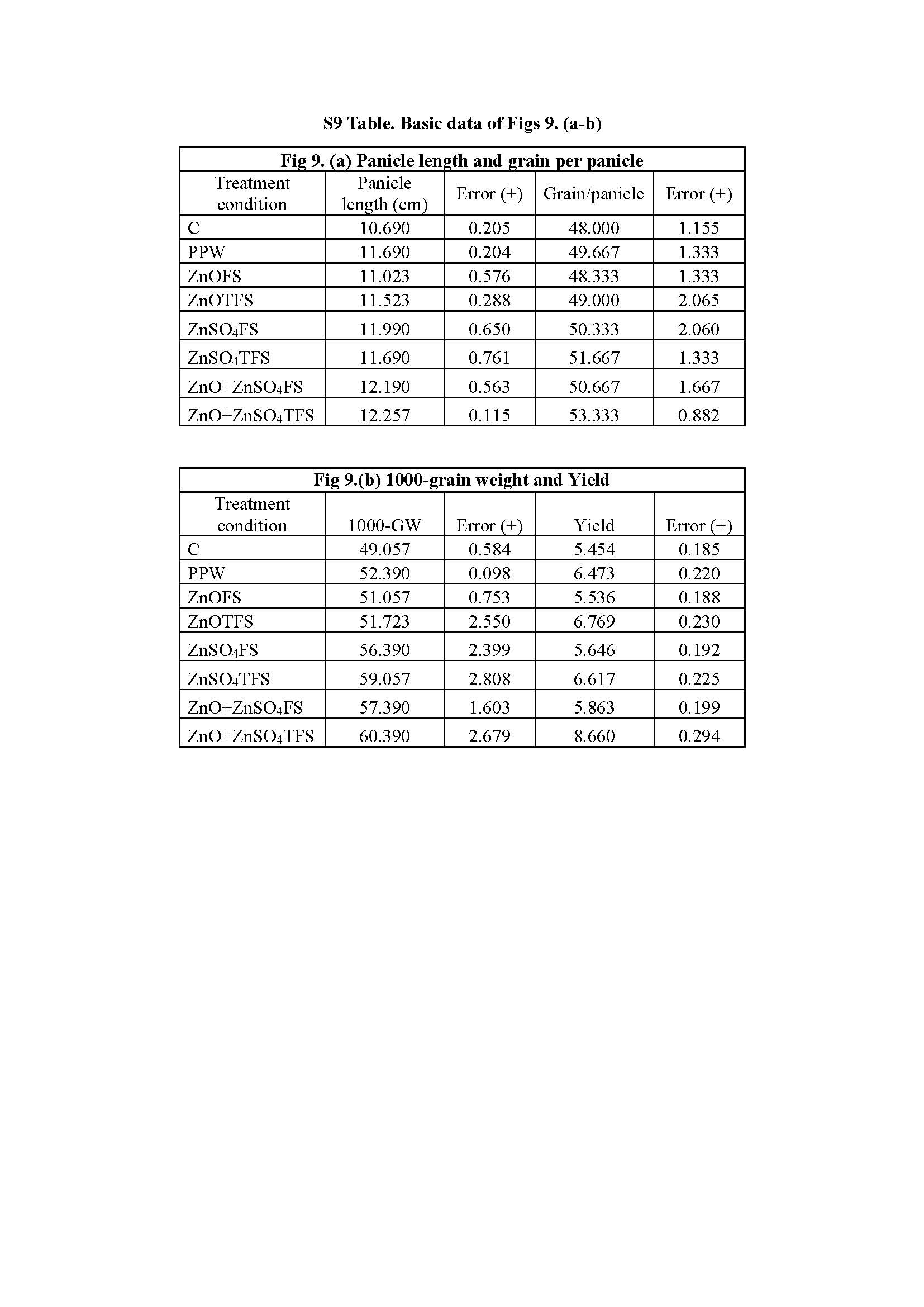

Supplement: S9 Table — (JPG) [file pone.0343231.s009.jpg]

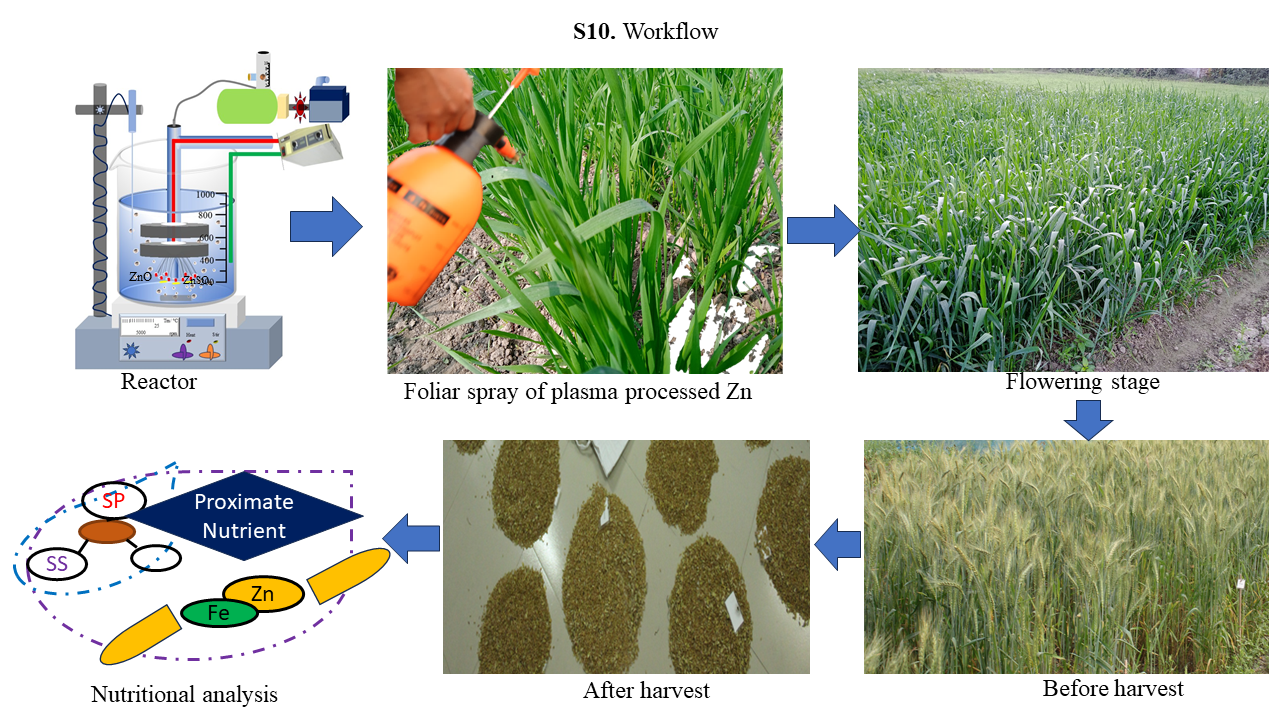

Supplement: S10 File — (TIF) [file pone.0343231.s010.tif]
